# S12-1 School-based interventions to promote physical activity amongst children and adolescents

**DOI:** 10.1093/eurpub/ckad133.058

**Published:** 2023-09-11

**Authors:** Chair(s):, Marie H Murphy, Angela Carlin

**Affiliations:** University of Edinburgh; Ulster University; Ulster University

## Abstract

**Background:**

Regular physical activity (PA) is associated with many benefits for physiological and mental wellbeing in children and adolescents. Despite this, many children and adolescents are failing to meet current PA guidelines. Given the age-related declines in PA as children move into adolescence, and then adulthood, the development of interventions to promote PA in this population is important. Schools have been identified as a key setting for the promotion of health-related behaviours, and young people spend a significant proportion of their waking day within the school setting.

**Aim:**

This symposium will engage researchers, practitioners, health care professionals and policy makers to discuss novel school-based interventions. The objectives of this symposium are to: (1) Disseminate the findings of school-based interventions across a number of countries (France, Ireland, United Kingdom, Spain); (2) Highlight cross-cultural challenges in the development and evaluation of school-based interventions, and (3) Outline how co-design and Patient and Public Involvement (PPI) have been utilised in the development of school-based interventions.

**Symposia presentations:**

1. The effectiveness of a peer-led school-based walking intervention on adolescent girls’ physical activity: the Walking In ScHools (WISH) study. This presentation will highlight findings from the first fully powered trial to investigate the effectiveness of a peer-led brisk walking intervention in adolescent girls, including primary and secondary outcome data, and process evaluation measures.

2. School-based physical activity promotion in a cross-cultural context: interventions from France and Spain. This presentation will highlight key learnings on how best to connect cross-cultural educational contexts for the promotion of physical activity and discuss the development of a cross-cultural intervention guide.

3. Co-production of the Move Well, Feel Good movement behaviours intervention. This presentation will describe the feasibility evaluation of the Move Well, Feel Good intervention. The Move Well, Feel Good intervention was developed using a co-design approach, and aims to enhance children’s mental health through motor competence and psychosocial development.

4. Physical activity trends among Second-level Active School Flag programme 2019-2021. This presentation will evaluate the impact of the Active School Flag programme and outline changes in physical activity levels over a three-year period.

